# Proteomic Analysis of the Hepatopancreas of Chinese Mitten Crabs (*Eriocheir sinensis*) Fed With a Linoleic Acid or α-Linolenic Acid Diet

**DOI:** 10.3389/fphys.2018.01430

**Published:** 2018-10-10

**Authors:** Banghong Wei, Zhigang Yang, Yongxu Cheng, Junyu Zhou, Hang Yang, Long Zhang, Xiaozhen Yang

**Affiliations:** ^1^Key Laboratory of Freshwater Aquatic Genetic Resources, Ministry of Agriculture, Shanghai Ocean University, Shanghai, China; ^2^Centre for Research on Environmental Ecology and Fish Nutrition, Ministry of Agriculture, Shanghai Ocean University, Shanghai, China; ^3^National Demonstration Center for Experimental Fisheries Science Education, Shanghai Ocean University, Shanghai, China

**Keywords:** *Eriocheir sinensis*, proteome, hepatopancreas, linoleic acid, α-linolenic acid, label-free quantification

## Abstract

As representatives of n-6 and n-3 fatty acids, many studies have analyzed the use of soybean oil and linseed oil rich in linoleic acid (18:2n-6, LA) and α-linolenic acid (18:3n-3, LNA) as better substitutes for fish oil. In aquatic animals, different dietary ratios of LA and LNA could have significant effects on growth, lipid metabolism, immune response, and reproduction. To assess the nutritive value of these two fatty acids in Chinese mitten crab (*Eriocheir sinensis*), we performed transcriptome analysis and label-free quantification proteomic analysis of the hepatopancreas from mitten crabs fed with LA or LNA diet. Parallel reaction monitoring was used to confirm the reliability of the proteomic analysis. A total of 186 proteins were differentially expressed with fold change ≥1.5 or ≤0.666. Among the 186 proteins, 116 were upregulated and 70 were downregulated in the LA than LNA. Most of these proteins participate in cellular process and metabolism process and have molecular functions such as binding and catalytic activity; the cellular component of these proteins are cell, cell part, membrane, and membrane part. A total of 18 proteins were identified to be related to lipid, carbohydrate, and protein metabolism, and they mainly participate in digestive enzyme activities, fatty acid transport, and glycolysis. Our results provide new insights for further investigation into the replacement of fish oil from mitten crabs with vegetable oils and enable us to better understand the different roles and nutrition value of LA and LNA in mitten crabs.

## Introduction

The Chinese mitten crab, *Eriocheir sinensis*, is a native freshwater crab species in China, and it is widely distributed in the streams and rivers along the eastern coast of China (Sui et al., 2009). Because of their nutritional value, flavor, and taste, mitten crabs have become one of the most important economic crab species in China. In 2016, 812,103 tons of mitten crabs was produced by the Chinese aquaculture industry, and ranked first among freshwater crustaceans (China Fisheries Yearbook, 2017). Mitten crabs have a high content of highly unsaturated fatty acids (HUFAs), such as eicosapentaenoic acid (EPA, 20:5n-3) and docosahexaenoic acid (DHA, 22:6n-3), in the hepatopancreas (Tang et al., 2013). To date, most studies have indicated that freshwater fish have the ability to synthesize HUFAs, and supplementation of linoleic acid (LA) and α-linoleic acid (LNA) in diets can satisfy the need for HUFAs (Tocher, 2010). In mitten crabs, several enzymes responsible for the desaturation and elongation of the polyunsaturated fatty acid have been identified, including three desaturases and two elongases, namely, fatty acyl Δ6-desaturase (Yang et al., 2013), fatty acyl-CoA Δ6-b desaturase (Yang et al., 2016b), acyl-CoA Δ9-desaturase (Guo et al., 2013), elongase of very long chain fatty acids (ELOVL) (Yang et al., 2016a), and ELOVL6 (Shi et al., 2016). However, characterization of these desaturases and elongases in mitten crab has shown that these enzymes are not directly involved in the synthesis of HUFAs such as EPA and DHA; therefore, the demands of HUFAs in mitten crabs are mainly provided by the diets.

Fish oil extracted from marine fish contains a high content of HUFAs, such as EPA and DHA, and it has been considered the best lipid source in aquatic feed (Sargent et al., 2002). However, a sharp decline in wild fisheries has significantly increased the price of fish oil, followed by an increase in feed production costs (Naylor et al., 2000; Tacon and Metian, 2009). To reduce the use of fish oil in the diets of mitten crabs, other lipid sources should be found to substitute fish oil. Lipid sources that contain LA and LNA (n-6 and n-3 fatty acid representatives, respectively), such as soybean oil and linseed oil, are considered to be the best substitutes for fish oil, and blends of these oils may be better than using the oils individually (Brown and Hart, 2011; Turchini et al., 2011). However, there is limited information on the optimal LA/LNA ratio in aquatic animal feeds. Many studies have investigated the effects of different dietary LA/LNA ratios in fish (Tian et al., 2016; Zeng et al., 2016a,b, 2017). However, to the best of our knowledge, little studies have investigated the optimal LA/LNA ratio in the diets of the mitten crab. To identify an optimal LA/LNA ratio for mitten crabs, we need to understand the different nutrition values of LA and LNA in mitten crabs.

With developments in mass spectrometry, proteomics has become a powerful tool for the analysis of multidisciplinary scientific questions (Megger et al., 2013). The label-free quantification proteomic approach allows simultaneous identification and quantification and is applicable to samples from any source (Turck et al., 2007; Li et al., 2012; Merl et al., 2012). In this study, the label-free quantification proteomic approach was used to analyze the effects of LA and LNA diets on mitten crabs and identify the differentially expressed proteins in the hepatopancreas of the crabs fed with LA and LNA diets.

Parallel reaction monitoring (PRM) is a new method in targeted mass spectrometry (Du et al., 2015), and it has been widely used to quantify and detect target proteins (Peterson et al., 2012; Tsuchiya et al., 2013). PRM analysis has been successfully used in the confirmation of proteomic analysis. PRM is more specific and sensitive than other monitoring methods, and comparing with western blotting, PRM could simultaneously quantify multiple proteins between two group (Gallien et al., 2014). In this study, PRM was used to confirm the proteomic analysis.

## Materials and Methods

### Ethics Statement

All the animal experiments were approved by the Animal Care Committee of Shanghai Ocean University, and were carried out in accordance with the principles of the Animal Care Committee of Shanghai Ocean University.

### Experimental Materials, Design, and Growth Performance

Two isonitrogenous and isolipidic purified experimental diets were formulated using different dietary lipid sources. The amounts of lipid and protein in the diets were 6 and 41%, respectively. The protein was provided by casein, and LA and LNA were the two lipid sources used in the study. The raw materials were blended and moistened with water and pelletized into 1.5 mm (diameter) pellets and stored at −20°C until use. The composition and formulation of the experimental diets are listed in **Table 1**.

**Table 1:** Composition and calculated proximate composition of the diets.

Ingredients (%)	LA	LNA
Casein	41	41
Cellulose	4	4
Wheat flour	28.65	28.65
Carboxymethylcellulose (CMC)	4	4
Yeast extract	5	5
Lysine	0.15	0.15
Glycine	0.5	0.5
Vitamin C (99.7%)	0.5	0.5
Vitamin E (97%)	0.1	0.1
Phospholipid (99%)	3	3
Cholesterol	0.5	0.5
Inositol	0.6	0.6
Choline chloride (50%)	1	1
Mineral premix^1^	3	3
Vitamin premix^2^	2	2
Linoleic acid (70%)	6	0
α-Linoleic acid (70%)	0	6
**Proximate composition (percentage dry weight)**		
Crude protein	39.33	39.27
Crude lipid	9.33	9.49
Ash	5.22	5.68

*^1^Mineral premix: 1 kg diet contains Ca(H_2_PO_4_)_2_, 10 g; MgSO_4_⋅7H_2_O, 2.4 g; KCl, 4.5 g; NaCl, 2.1 g; FeSO_4_⋅H_2_O, 155 mg; CuSO_4_⋅5H_2_O, 40 mg; ZnSO_4_⋅H_2_O, 80 mg; MnSO_4_⋅H_2_O, 30 mg; KI, 11.7 mg; CoCl_2_⋅6H_2_O, 4.8 mg; Na_2_SeO_3_, 2.4 mg. ^2^Vitamin premix: 1 kg diet contains vitamin A, 10,000 IU; vitamin D, 2500 IU; vitamin K, 64 mg; thiamin, 60 mg; riboflavin, 250 mg; pyridoxine, 60 mg; calcium pantothenate, 240 mg; niacin, 60 mg; folic acid, 12 mg; biotin, 50 mg; cyanocobalamin, 4 mg.*

All the mitten crabs used in the study were obtained from the Chongming Research Base of Shanghai Ocean University. All the crabs were separately acclimated in a single plastic box (36 cm × 18 cm × 18 cm) for a week. During the acclimation, all the crabs were fed with a commercial feed. After acclimation, 48 active male crabs with intact appendages were selected and randomly stocked into 48 plastic boxes (36 cm × 18 cm × 18 cm). Then, the 48 crabs were classified into two groups (24 crabs in each group) and fed with either the LA or LNA diet. Before the feeding experiment, all the crabs in each group were weighted, the carapace length and width were measured. From **Table 2**, the initial weight, carapace length, and width of crabs in each group were same.

**Table 2:** Effects of different fatty acids diets on growth performance of juvenile Chinese mitten crab, *Eriocheir sinensis*.

	Groups	
	LA	LNA
Survival rate (%)	91.67	91.67
Initial weight (g, *n* = 24)	1.66 ± 0.13	1.69 ± 0.11
Initial carapace length (mm, *n* = 24)	13.82 ± 0.42	13.94 ± 0.29
Initial carapace width (mm, *n* = 24)	15.72 ± 0.56	15.73 ± 0.43
Final weight (g, *n* = 22)	4.47 ± 1.23	4.59 ± 1.25
Final carapace length (mm, *n* = 22)	18.49 ± 1.67	18.58 ± 1.98
Final carapace width (mm, *n* = 22)	20.18 ± 1.76	20.22 ± 1.97
Weight gain (*n* = 22, %)	169.45 ± 71.93	172.86 ± 81.24
Specific growth rate (*n* = 22, %/day)	0.93 ± 0.25	0.93 ± 0.29

The feeding experiment lasted for 107 days. During the experiment, the crabs were fed daily at 13:30. The experimental diets were administered at 5% body weight of the crabs. Leftover feed was removed using a siphon tube at 15:30. Throughout the experiment, all the boxes were aerated to maintain the dissolved oxygen at >5 mg/L. The photoperiod was 12 h light:12 h dark. Water in each box was exchanged once daily (1/3 to 1/2 of the tank volume). The water parameters in each box were maintained within the following ranges: temperature, 24.5–30°C; pH, 8.0 ± 0.4; and total ammonia nitrogen, <0.01 mg/L. At the end of feeding experiment, the weight, carapace length, and width of crabs in each group were also weighted and measured. The weight gain and specific growth rate were calculated in the following methods:

Weight gain (%)=(final weight−initial weight)/initial weight×100

Specific growth rate (SGR, %/day)=(Ln final weight−Ln initial weight)×100/days

### Sample Collection and Preparation

At the end of the feeding trial, all the crabs were fasted for 24 h. A total of six crabs that had the average weight were selected for the use of the proteomic analysis with three crabs in each group, and the hepatopancreas was collected from the three crabs in sterile centrifuge tubes and immediately frozen in liquid nitrogen. Then, the samples were stored at −80°C until use. Protein extraction was performed using the Mammalian Tissue Total Protein Extraction Reagent (AP0601-50) from Bangfei Bioscience. Before protein extraction, the hepatopancreas from three crabs in each group were pooled into one sample, and then two pooled hepatopancreas samples were ground in liquid nitrogen and rinsed twice with 1 mL of phosphate-buffered saline. Then, the samples were homogenized in the protein lysate containing a protease inhibitor. After centrifugation for 10 min, the supernatant was collected and stored at −80°C for further analysis.

### Protein Quantification and Proteolysis

The extracted proteins were quantified using the Bradford method. The calibration curve was established using protein standards. Protein quantification was performed using the calibration curve and optical density values of the protein samples. After quantification, 60 μg of the protein samples was digested using trypsin (Promega, United States) in a ratio of protein:trypsin at 50:1. The protein samples were digested at 37°C for 12–16 h.

### Liquid Chromatography-Tandem Mass Spectrometry Analysis

After proteolysis, two peptides samples were obtained, then the peptides in each group were divided into triplicate for the proteomic analysis to minimize equipment error. Gradient elution and separation were performed using Nano high-performance liquid chromatography Ultimate 3000 (Thermo Fisher Scientific, United States) with solutions A and B (A: 0.1% formic acid in water, B: 80% acetonitrile with 0.1% formic acid in water). Mass spectrometry analysis was performed using Q Exactive HF (Thermo Fisher Scientific, United States) coupled to the Nano high-performance liquid chromatography Ultimate 3000. Full MS spectra were obtained with a scan range of 300–1400 m/z, resolution of 120,000, automatic gain control target value of 3e^6^, maximum ion accumulation time of 80 ms, and one number of scan range. The MS2 scan was set at a resolution of 15,000, automatic gain control target value of 5e^4^, maximum ion accumulation time of 45 ms, and isolation window of 1.6 m/z.

### Protein Identification and Quantification

As a transcriptome analysis was performed before the proteome analysis, the mass spectra was searched against the database from the transcriptome analysis by using Proteome Discoverer, version 2.0 (Thermo Fisher Scientific, United States) and in-house Mascot, version 2.2 (Matrix Science, United Kingdom). The search results were then filtered using a cutoff of 1% for the peptide false identification rate. Label-free quantification was used for quantification of the proteins. For quantitative changes, a cutoff of ≥1.5 or ≤0.666-fold change and *p*-value (*t*-test) <0.05 were set for differentially expressed proteins.

### Bioinformatic Analysis

In this study, bioinformatic analysis of the proteome was performed by matching the accessions between the proteome and transcriptome. For the transcriptome analysis, the assembled unigenes were aligned with the non-redundant database (Nr), Gene Ontology (GO), euKaryotic Ortholog Groups (KOG), Kyoto Encyclopedia of Genes and Genomes (KEGG) by using BlastX to obtain annotation of the genes. Cluster enrichment of GO terms and KEGG pathways were analyzed using hypergeometric distribution.

### PRM Analysis

Parallel reaction monitoring analysis was used to confirm the label-free quantification proteome analysis. Twelve proteins were selected for the PRM analysis. Protein extraction, proteolysis, mass spectrometry analysis, and protein identification were performed according to the methods described previously. Skyline 3.6 software was used for analysis of the raw data of PRM, and quantification of the selected proteins was performed using MSstat. The fold changes in the LNA and LA groups between PRM and label-free quantification proteome were compared for confirmation.

### Statistical Analysis

Statistical analysis was performed using SPSS Statistics V22.0 (IBM Corporation, NY, United States). Growth performance was presented as means ± SD and was analyzed by Student’s *t*-test. Data were considered to be statistically significant when *P* < 0.05.

## Results

### Growth Performance

After the feeding experiment, 22 crabs in each group were survived with the survival rate of 91.67% (**Table 2**). And the final weight, carapace length, width, weight gain, and specific growth rate were same between the crabs fed with LA or LNA (**Table 2**).

### Protein Identification

**FIGURE 1:**
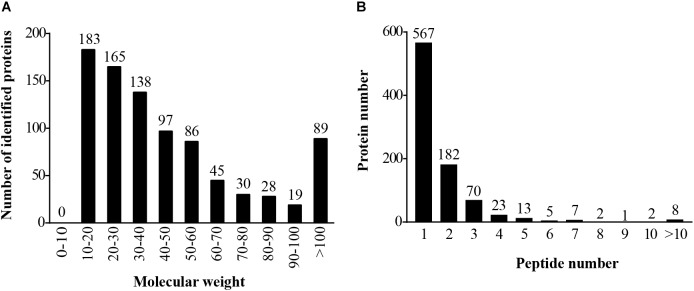
Identification and analysis of proteins in the hepatopancreas of mitten crabs fed with LA and LNA diets. **(A)** Distribution of the identified proteins among different molecular-weight classes. **(B)** Distribution of proteins containing different numbers of identified peptides.

The mass spectrometry proteomics data have been deposited to the ProteomeXchange Consortium via the PRIDE partner repository with the dataset identifier PXD009864. The LC-MS/MS generated a total of 1,530 unique peptides, and 880 proteins were identified (**Supplementary Table S1**). The number of identified proteins with molecular weight in the range of 10–20 kD, 20–30 kD, 30–40 kD, 40–50 kD, and 50–60 kD was 183 (20.80%), 165 (18.75%), 138 (15.68%), 97 (11.02%), and 86 (9.77%), respectively, whereas 23.98% proteins had molecular weight >60 kD (**Figure 1A**). Most of the identified proteins (567) had unique peptides, and about 14.89% of the identified proteins had three or more peptides (**Figure 1B**). About 21.48% of the identified proteins showed more than 10% peptide coverage.

### Differentially Expressed Proteins

Because a cutoff of ≥1.5 or ≤0.666-fold change was set for differentially expressed proteins, 186 proteins were found to be differentially expressed between the LA and LNA groups (**Supplementary Table S2**). When compared with the LA group, 116 proteins were downregulated and 70 proteins were significantly upregulated in the LNA group. Nr annotation of the differentially expressed proteins showed that the five most significantly downregulated proteins were elongation factor-1 alpha, histone H4, glyceraldehyde-3-phosphate dehydrogenase, nucleophosmin, and alpha-enolase, and the five most significantly upregulated proteins were fatty-acid-binding protein (FABP), RNA polymerase II transcription elongation factor, vitelline membrane outer layer 1, eukaryotic translation initiation factor 5A, and ribosomal protein S3a.

### Functional Analysis of the Differentially Expressed Proteins

**FIGURE 2:**
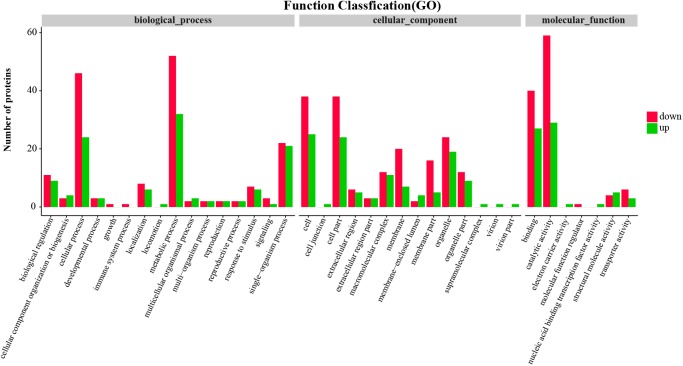
Gene Ontology (GO) function classification of differentially expressed proteins in the hepatopancreas of mitten crabs fed with LA and LNA diets.

Function annotation of the differentially expressed proteins was performed to understand the function and bioprocess of the differentially expressed proteins. GO annotation of the differentially expressed proteins showed that both upregulated and downregulated proteins were mainly categorized into metabolism process and cellular process in biological processes, cell and cell part in cellular component, and catalytic activity and binding in molecular function (**Figure 2**).

**FIGURE 3:**
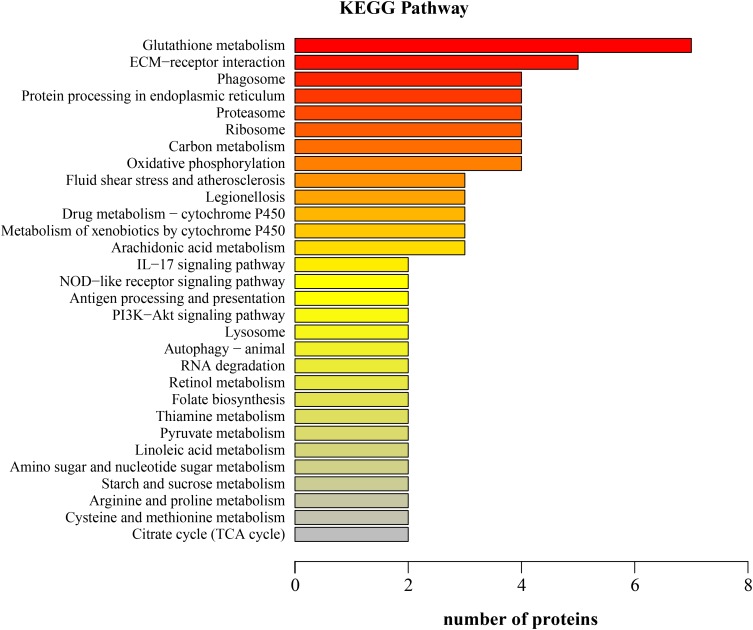
Kyoto Encyclopedia of Genes and Genomes (KEGG) function classification of the downregulated proteins in the hepatopancreas of mitten crabs fed with LA and LNA diets (LNA vs. LA).

Kyoto Encyclopedia of Genes and Genomes annotation showed that the downregulated proteins in the LNA participated in 60 KEGG pathways; the top five pathways were glutathione metabolism, ECM-receptor interaction, phagosome, protein processing in endoplasmic reticulum, and proteasome (**Figure 3**). The differentially expressed proteins that participated in these pathways were glutathione *S*-transferase, thioredoxin domain-containing protein, aminopeptidase, prostaglandin D synthase, collagen, laminin subunit, integrin, cathepsin L protein, V-type proton ATPase subunit G, calnexin, heat shock protein, and 26S protease regulatory subunit.

**FIGURE 4:**
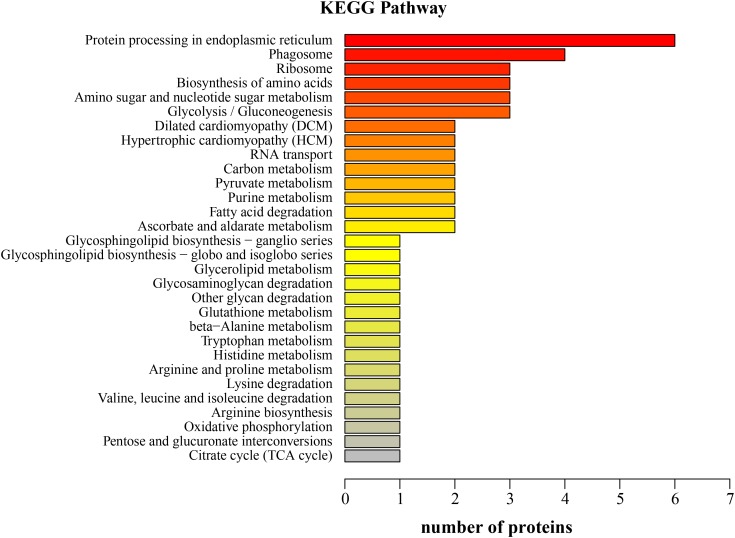
Kyoto Encyclopedia of Genes and Genomes function classification of the upregulated proteins in the hepatopancreas of mitten crabs fed with LA and LNA diets (LNA vs. LA).

Sixty-four KEGG pathways were annotated for the 70 upregulated proteins in the LNA. The top five KEGG pathways were protein processing in endoplasmic reticulum, phagosome, ribosome, biosynthesis of amino acids, and amino sugar and nucleotide sugar metabolism (**Figure 4**). The relevant proteins were protein-disulfide isomerase, translocon-associated protein subunit alpha, B-cell receptor-associated protein, actin, calreticulin, 60S ribosomal protein, ribosomal protein S3a, glyceraldehyde 3-phosphate dehydrogenase, pyruvate kinase 3, chitinase 1, *N*-acetylneuraminate lyase, and β-hexosaminidase subunit beta.

### Differentially Expressed Proteins Related to Metabolism

**FIGURE 5:**
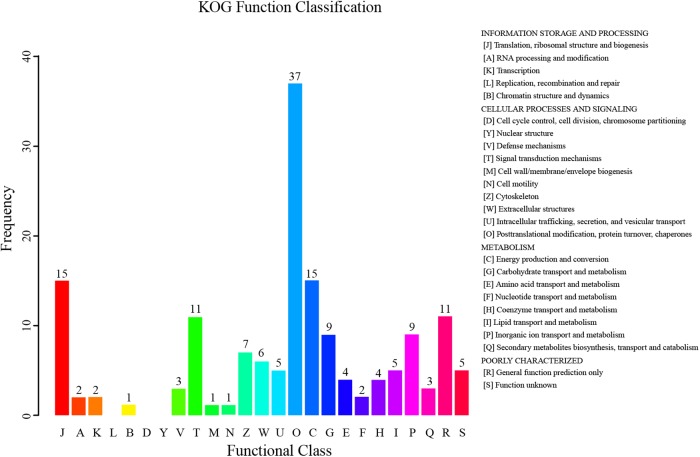
euKaryotic Ortholog Groups (KOG) function classification of differentially expressed proteins in the hepatopancreas of mitten crabs fed with LA and LNA diets.

According to the KOG annotation of the differentially expressed proteins, many proteins related to metabolism were identified (**Figure 5**). A total of nine proteins were related to carbohydrate transport and metabolism. Metabolism of proteins and lipids was also significantly changed by the dietary fatty acids, as four and five proteins were related to amino acid transport and metabolism and lipid transport and metabolism, respectively. The relevant proteins are listed in **Table 3**.

**Table 3:** Differentially expressed proteins related to metabolism in the mitten crabs fed with LA or LNA diet (LNA vs. LA).

Accession number	Protein name	Fold change	*P*-value
**Carbohydrate transport and metabolism**			
TRINITY_DN88199_c4_g1	UDP-glycosyltransferase	3.577438	0.040568455
TRINITY_DN63735_c15_g2	Alpha-amylase	6.769554	6.60E-05
TRINITY_DN44590_c0_g2	Glyceraldehyde 3-phosphate dehydrogenase	3.757887	0.034006103
TRINITY_DN96303_c0_g8	Pyruvate kinase	4.987022	3.94E-05
TRINITY_DN118188_c0_g1	Enolase	0.025047	0.013138905
TRINITY_DN98106_c1_g6	Glucose-6-phosphate isomerase	0.45506	0.016351927
TRINITY_DN89776_c14_g1	Chitinase	1.598054	0.005352149
TRINITY_DN72125_c0_g1	Predicted glyoxalase	0.256407	0.037162005
TRINITY_DN92506_c5_g1	Beta-*N*-acetylhexosaminidase	6.350409	0.019840784
**Amino acid transport and metabolism**			
TRINITY_DN97960_c2_g43	Puromycin-sensitive aminopeptidase	2.267724	0.021308279
TRINITY_DN87925_c1_g1	Glutamate dehydrogenases	0.518076	0.030079441
TRINITY_DN94282_c1_g28	Aminoacylase	2.627925	0.006488639
TRINITY_DN94106_c129_g1	Trypsin	1.546861	0.031488187
**Lipid transport and metabolism**			
TRINITY_DN97067_c1_g13	Predicted phosphate acyltransferases	0.319421	0.000336802
TRINITY_DN94524_c11_g41	Peroxisomal phytanoyl-CoA hydroxylase	3.463067	0.039780981
TRINITY_DN86179_c18_g1	Fatty-acid-binding protein FABP3	85.67045	0.015058246
TRINITY_DN98078_c88_g1	Predicted lipoprotein	0.428003	0.002560041
TRINITY_DN85667_c0_g1	Long-chain fatty acid transport protein	0.479258	0.021174358

### PRM Analysis

**FIGURE 6:**
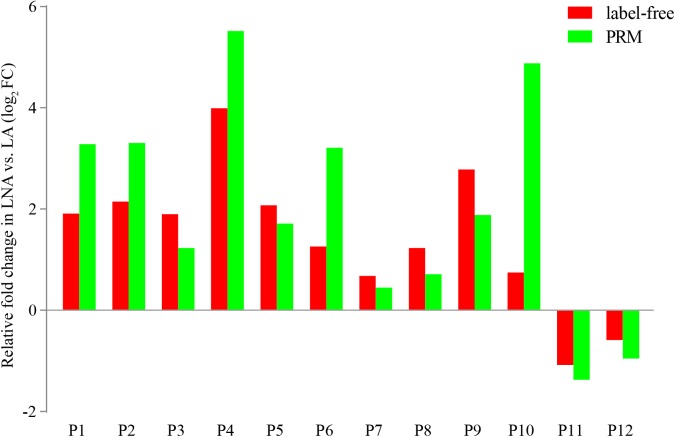
Confirmation of differentially expressed proteins detected in the proteome analysis by using PRM analysis. P1: glyceraldehyde 3-phosphate dehydrogenase, P2: hemocyanin, P3: 60S ribosomal protein L5, P4: eukaryotic translation initiation factor, P5: alpha tubulin, P6: adenosine kinase, P7: chitinase, P8: spectrin alpha chain, P9: calreticulin, P10: hemocyanin subunit 6, P11: endo-beta-1,4-glucanase, P12: laminin subunit alpha.

Parallel reaction monitoring analysis was used to confirm the differentially expressed proteins in the proteome. Twelve differentially expressed proteins with a unique peptide were selected for the PRM analysis, namely, glyceraldehyde 3-phosphate dehydrogenase, hemocyanin, 60S ribosomal protein L5, eukaryotic translation initiation factor 5A, tubulin, adenosine kinase 1, chitinase, spectrin alpha chain, calreticulin, hemocyanin subunit 6, endo-beta-1,4-glucanase, and laminin subunit alpha. The fold changes in these proteins were consistent with the results of the label-free quantification proteome analysis (**Figure 6**).

## Discussion

To the best of our knowledge, the mitten crab has limited ability to synthesize HUFAs by LA or LNA, as a results, the HUFAs, such as EPA and DHA, are necessary in the diet of mitten crabs (Wu et al., 2007). The higher contents of EPA and DHA in mitten crabs (Chen et al., 2007), as well as the important roles of EPA and DHA in humans, have attracted a lot of research interest in the nutritional value of EPA and DHA in mitten crabs. The beneficial effects of LA and LNA have been ignored as the importance of EPA and DHA. In this study, we performed label-free quantification proteomic analysis to investigate the nutritional value of LA and LNA in the mitten crab.

The hepatopancreas plays an important role in the mitten crab; it is a major digestive gland and has a role in the synthesis and secretion of digestive enzymes as well as digestion and absorption of nutrients (Jiang et al., 2009). The hepatopancreas is also responsible for metabolism in crustaceans (Rosas et al., 1995; Li et al., 2008). Proteomics is a new system biology technique that theoretically presents all the proteins in an organism (Anderson and Anderson, 1998), and comparative proteomic analysis can identify the proteomic differences caused by different factors (Lemos et al., 2010). Thus, comparative proteomic analysis of the hepatopancreas is a practicable method for the analysis of the nutrition value of LA and LNA in the mitten crab.

In rates, LA and LNA have been observed to ameliorate the metabolic syndrome induced by high-fructose and high-fat diets (Zhang et al., 2016). Dietary LA and LNA could have significant effects on carbohydrate and lipid metabolism, and the effects depend on the dose of LA and LNA rather than the ratio; in other words, the roles of LA and LNA are different (Zhang et al., 2016). In the present study, the same results were obtained, several proteins that participated in the metabolism and transport of three major nutrients were significantly differently expressed in the crabs fed with LA and LNA diets.

### Lipid Metabolism

A standard metabolism analysis has shown that lipid is the main source for energy metabolism in the mitten crab, followed by carbohydrate and protein (Wen et al., 2001); this indicated the important role of lipid metabolism in the mitten crab. In the present study, five proteins relative to lipid metabolism in the crabs fed with LA and LNA diets were significantly changed. The level of FABP3 was significantly higher in the crabs fed with LNA than in those fed with LA. FABPs belong to a gene family that helps in the transport of long-chain fatty acids across the plasma membrane (Kaikaus et al., 1990). FABP3 is responsible for the transport of fatty acids for β-oxidation (Jordal et al., 2006). Besides FABP3, two proteins, namely, a lipoprotein and long-chain fatty acid transport protein, were significantly changed in the LA and LNA groups. The lipoprotein and long-chain fatty acid transport protein have roles in the transport of lipid and fatty acid, respectively (Schaffer and Lodish, 1994; Rodenburg and Van der Horst, 2005). An optimal ratio of LA/LNA could inhibit fat deposition (Zeng et al., 2016b). In the present study, we observed that the effects of LA and LNA on fat deposition were dependent on two different parameters: LNA increased fatty acid transport for β-oxidation, whereas LA increased the levels of the lipoprotein and long-chain fatty acid transport protein to decrease lipid deposition. Because of the different roles of LA and LNA in lipid metabolism, an optimal blending of LA and LNA might be better than the use of either LA or LNA alone (Brown and Hart, 2011; Turchini et al., 2011).

### Carbohydrate Metabolism

Chitin is a typical polysaccharide and exists in the cuticles of mitten crabs (Kurita, 2006). At the time of molting, the exoskeleton composed of chitin is cast off (Kono et al., 1995). Chitinase, which hydrolyzes chitin, has an important role in the molting of the mitten crab (Dahiya et al., 2006; Yao et al., 2015). Previous studies have indicated that the expression of chitinase is regulated by many factors, such as salinity (Zhang et al., 2015). However, to our knowledge, no study has investigated the effects of dietary fatty acids on chitinase. In the present study, the level of chitinase was significantly higher in the LNA group than in the LA group, which may indicate that LNA has a role in the regulation of molting in the mitten crab. The effects of LA and LNA on carbohydrate metabolism, which has a role in type 2 diabetes mellitus, have been thoroughly investigated in humans; however, further studies are needed to elucidate the effects of LA and LNA on glucose metabolism (Cabout et al., 2017). In this study, several proteins that participate in glycolysis were significantly changed in the LA and LNA groups, which indicates that LA and LNA have different roles in glucose metabolism in the mitten crab. Glycolysis is a universal pathway that exists in living cells. The end product of glycolysis is lactate under anaerobic conditions, while pyruvate is formed in the presence of oxygen and is then oxidized to CO_2_ and H_2_O (Akram, 2013). Glycolysis requires three key enzymes, one of which is pyruvate kinase (Li et al., 2015). The higher level of pyruvate kinase in the LNA group may indicate the more important role of LNA than LA in glycolysis. However, the levels of other enzymes that participate in glycolysis, such as enolase and glucose-6-phosphate isomerase, were higher in the LA group, suggesting that LA is also an important factor for the regulation of glycolysis in the mitten crab. Our results indicate that blends of LA and LNA may be better than the individual supplements. Alpha-amylase was also differentially expressed in the LA and LNA groups; it was expressed significantly higher in the LNA group than in the LA group. In grass carp, the activity of amylase could be significantly increased by increasing the LNA/LA ratio to a certain degree (Zeng et al., 2016b). On the basis of our results, we can conclude that LNA may have a more important role in the acceleration of amylase than LA. For omnivorous species, amylase is important for digestion. A previous study showed that mitten crabs fed with a linseed oil diet (2.03) had a lower feed coefficient than mitten crabs fed with a soybean oil diet (2.46) (Wang et al., 2017). We speculate that improvement of the feed coefficient in the linseed oil group is mainly due to the higher abundance of amylase, as well as trypsin, attributable to LNA.

### Protein Metabolism

Besides trypsin, three proteins were significantly changed between the LNA and LA groups. In the mitten crab, glutamate dehydrogenases were isolated in 2012, and they have been proven to have an important role in controlling osmoregulation (Wang et al., 2012). Puromycin-sensitive aminopeptidase is an enzyme with roles in a number of physiological processes, such as normal cellular protein turnover, cell cycle regulation, processing of antigenic peptides for display on class I MHC and degradation of neuropeptides (Goldberg and Rock, 1992; Stoltze et al., 2000; Schulz et al., 2001; Lilley et al., 2005). In the present study, the levels of glutamate dehydrogenases were significantly higher in the LNA group than in the LA group, while puromycin-sensitive aminopeptidase level was higher in the LA group. However, further studies are required to investigate the effects of LNA and LA on osmoregulation and the above-mentioned physiological processes.

## Conclusion

The present study demonstrated that dietary LA and LNA have different nutrition values in the mitten crab, particularly with respect to digestive enzyme activities, fatty acid transport, and glycolysis. The different roles of LA and LNA in mitten crabs are complementary, and blending LA and LNA may have better effects than the individual supplements. Our results provide new insights for the replacement of fish oil from mitten crabs with vegetable oils and enable us to better understand the different roles and nutrition value of LA and LNA in mitten crabs.

## Author Contributions

BW, ZY, XY, and YC conceived and designed the study. BW, JZ, HY, and LZ collected the samples. BW and ZY acquired and analyzed the data. BW and ZY drafted the manuscript. All the authors have read and approved the final manuscript.

## Conflict of Interest Statement

The authors declare that the research was conducted in the absence of any commercial or financial relationships that could be construed as a potential conflict of interest.

## Supplementary Material

TABLE S1 Proteins identified in the hepatopancreas of mitten crabs fed with LA and LNA diets by using the label-free quantification proteome approach.

TABLE S2 Differentially expressed proteins identified in the hepatopancreas of mitten crabs fed with LA and LNA diets by using the label-free quantification proteome approach.
